# American Journal of Science and Art

**Published:** 1845-09

**Authors:** 


AMERICAN JOURNAL OF SCIENCE AND ART.

This work will have reached the 50th volume at the end of the present year, when it is the purpose of the editors to commence a new series. Now is the time for new subscribers to begin. The work is one which has reflected great credit upon American science, and deserves well of the scientific men of the country. It is high time that our profession was doing more for general science, and the Journal we are recommending would put its members in the way to it. Great would be the improvement seen in a few years if it could find its way into every village and neighborhood in the country, and draw medical men to it as constant readers—great the improvement in taste, and the extension of useful knowledge. May we not hope, that since the new series is about to commence, there will be a new impulse given to its subscription-list, and that for the future its patronage will be equal to its merits? Hitherto that has not been the case. It has barely lived, when the labors bestowed upon it by its gifted and admirable senior editor, Prof. Silliman, ought to have secured it most ample support. Professor S. is now assisted by his son, who, as a chemist and philosopher, promises to be worthy of his distinguished father. We shall rejoice, not more on account of the worthy editors, than for the sake of science, to learn that the American Journal of Science and Art has doubled its subscription, as it ought, with the beginning of the new series.

